# Antibiotic prescribing for acute infections in synchronous telehealth consultations: a systematic review and meta-analysis

**DOI:** 10.3399/BJGPO.2021.0106

**Published:** 2021-11-10

**Authors:** Mina Bakhit, Emma Baillie, Natalia Krzyzaniak, Mieke van Driel, Justin Clark, Paul Glasziou, Christopher Del Mar

**Affiliations:** 1 Institute for Evidence-Based Healthcare, Faculty of Health Sciences and Medicine, Bond University, Robina, Australia; 2 Primary Care Clinical Unit, Faculty of Medicine, The University of Queensland, Brisbane, Australia

**Keywords:** anti-bacterial agents, COVID-19, drug resistance, microbial, primary health care, remote consultation, respiratory tract infections, SARS-CoV-2, telemedicine, urinary tract infections

## Abstract

**Background:**

Antibiotic overprescribing is a major concern that contributes to the problem of antibiotic resistance.

**Aim:**

To assess the effect on antibiotic prescribing in primary care of telehealth (TH) consultations compared with face-to-face (F2F).

**Design & setting:**

Systematic review and meta-analysis of adult or paediatric patients with a history of a community-acquired acute infection (respiratory, urinary, or skin and soft tissue). Studies were included that compared synchronous TH consultations (phone or video-based) to F2F consultations in primary care.

**Method:**

PubMed, Embase, Cochrane CENTRAL (inception–2021), clinical trial registries and citing–cited references of included studies were searched. Two review authors independently screened the studies and extracted the data.

**Results:**

Thirteen studies were identified. The one small randomised controlled trial (RCT) found a non-significant 25% relative increase in antibiotic prescribing in the TH group. The remaining 10 were observational studies but did not control well for confounding and, therefore, were at high risk of bias. When pooled by specific infections, there was no consistent pattern. The six studies of sinusitis — including one before–after study — showed significantly less prescribing for acute rhinosinusitis in TH consultations, whereas the two studies of acute otitis media showed a significant increase. Pharyngitis, conjunctivitis, and urinary tract infections showed non-significant higher prescribing in the TH group. Bronchitis showed no change in prescribing.

**Conclusion:**

The impact of TH on prescribing appears to vary between conditions, with more increases than reductions. There is insufficient evidence to draw strong conclusions, however, and higher quality research is urgently needed.

## How this fits in

Acute infections are commonly treated with antibiotics, which adds to the problem of antibiotic resistance. Owing to the coronavirus pandemic (COVID-19), there was a shift towards remote consultations to decrease the risk of infection and transmission. However, it is not clear if TH consultations are contributing to antibiotic overuse or not. This study assessed the effect on antibiotic prescribing in primary care of TH consultations compared with F2F for acute infections. The impact of TH on prescribing appears to vary between conditions, with more increases than reductions. However, there is insufficient evidence to draw strong conclusions, and higher quality research is urgently needed.

## Introduction

Antibiotic overprescribing is a major concern that contributes to the problem of antibiotic resistance.^1^ In Australia, >41% of the population received at least one antibiotic in 2017,^2^ and 80% of antibiotic prescriptions occurred in primary care.^3^

In primary care, antibiotics are frequently prescribed for self-limiting acute respiratory infections such as middle ear infections, acute bronchitis, and sore throat,^4^ where antibiotics are of little benefit^5–8^ and may cause harms (for example, vomiting, diarrhoea, and rash).

Before the COVID-19 pandemic, several strategies (such as delayed prescribing) and campaigns (such as the Choosing Wisely campaigns) aimed to reduce antibiotic prescribing. In Australia, antibiotics are usually prescribed in an F2F consultation with GPs. However, remuneration for TH was introduced during the COVID-19 pandemic and many clinicians have shifted to deliver patient care remotely to decrease the risk of transmission. This change in mode of delivery may influence prescribing.

A recent systematic review by Han *et al* ^9^ found insufficient evidence to draw confident conclusions on the effect of TH consultations on antibiotic prescribing. This review has several limitations, mainly related to the search strategy (it included studies for both synchronous and asynchronous TH consultations) and the method of analysis of the included studies, which hindered the interpretation of the impact of TH on antibiotic prescribing. By contrast, the present systematic review focused only on synchronous TH consultations, which are more comparable to F2F consultations. The search strategy included broader keywords and MeSH Database terms to find any relevant studies, and employed a more detailed analysis subgrouped by the different conditions.

This systematic review aimed to identify and synthesise studies that have assessed the effect of synchronous TH consultations on antibiotic prescribing compared with F2F clinical encounters.

## Method

This systematic review is reported following the Preferred Reporting Items for Systematic Reviews and Meta-Analyses (PRISMA) statement.^10^ The protocol was developed prospectively and registered on the International prospective register of systematic reviews (PROSPERO) registration number CRD42021239164. The 2 week systematic review (2weekSR) processes were followed.^11^

### Eligibility criteria

#### Participants

Studies of adult or paediatric patients with a history of a community-acquired acute infection (respiratory, urinary, or skin and soft tissue) were included. Studies of hospitalised patients or patients with chronic infections were excluded.

#### Interventions

Studies of any type of synchronous TH consultations (phone or video-based) were included. Studies that reported the use of asynchronous TH consultations (text-based or web-based with automated feedback) were excluded. Studies with TH consultations combined with an education component were excluded unless the education component was given to both groups.

#### Comparators

Studies were included that compared TH consultations with the usual F2F consultations.

#### Outcomes (primary and secondary)

The primary outcome was the number of antibiotic prescriptions in each type of consultation.

The secondary outcomes were follow-up visit rates, testing rates or number of samples sent to the laboratory, any reported adverse events (AE), hospitalisation, and associated costs.

#### Study design

RCTs of any design (for example, parallel, cluster, crossover) and any type of observational studies were included. Reviews of primary studies (for example, systematic reviews, or literature reviews) were excluded.

### Search strategies

#### Database search

PubMed, Embase, and Cochrane CENTRAL were searched from inception to 23 February 2021. The search string was designed in PubMed, then translated for use in the other databases using the Polyglot Search Translator.^12^ The complete search strings for all databases are provided in Supplementary Box S1.

Clinical trial registries were searched on 2 March 2021 via Cochrane CENTRAL, which includes the WHO International Clinical Trials Registry Platform (ICTRP) and clinicaltrials.gov. Preprint articles were also searched for through the Europe PMC database.

On 1 March 2021, a backwards (cited) and forwards (citing) citation analysis was conducted in Scopus on the included studies identified by the database searches. These were screened against the inclusion criteria.

No restrictions by language or publication date were imposed. Publications that were published in full were included. Publications available as abstract only (for example, conference abstract) were included if they had a clinical trial registry record, or other public report, with the additional information required for inclusion. Publications available as abstract only (for example, conference abstracts) were excluded, unless additional information was available.

### Study selection and screening

**Figure 1.**
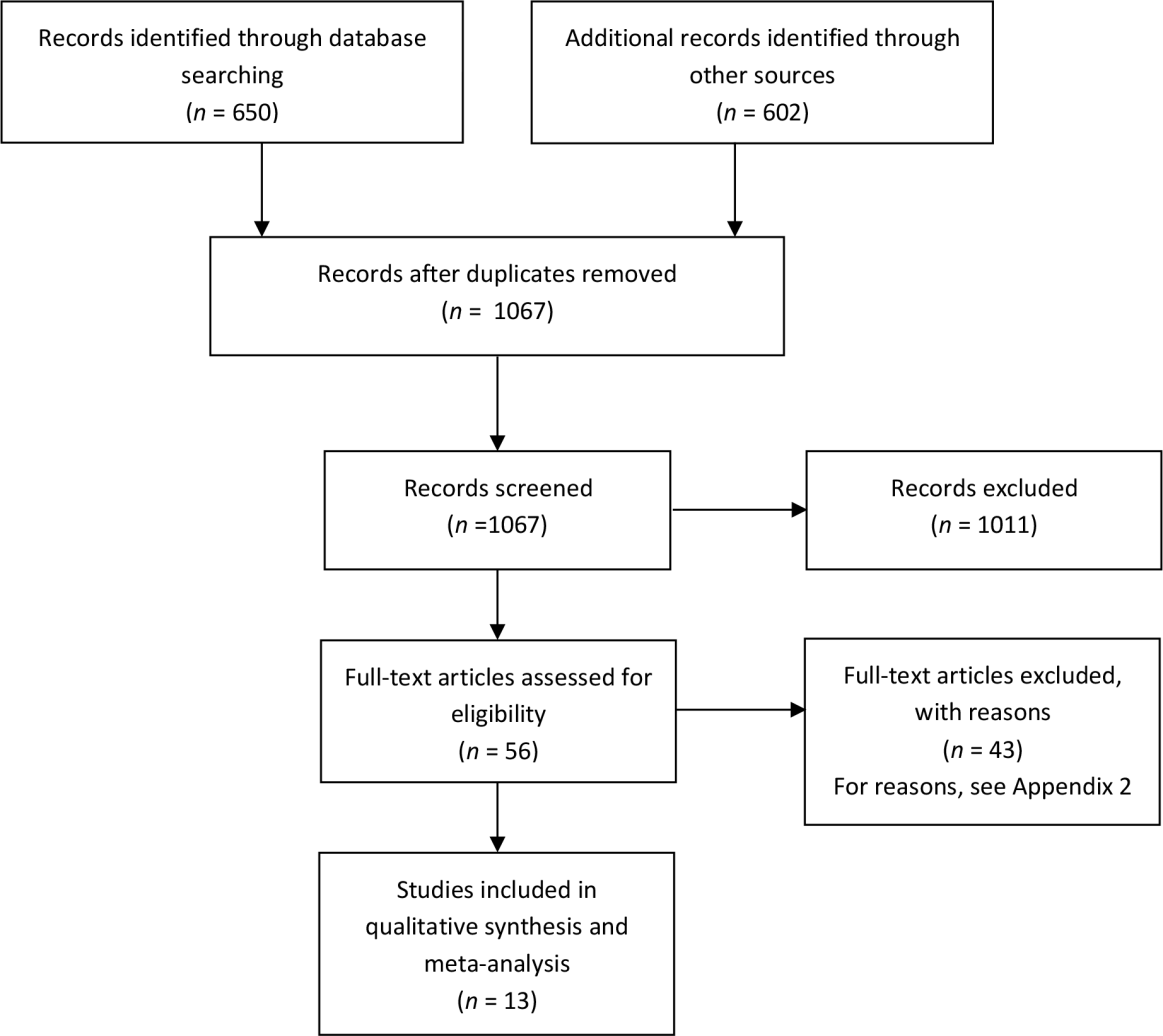
PRISMA flow diagram^10^

Two review authors (MB, and EB or NK) independently screened the titles and abstracts for inclusion against the inclusion criteria. One author (JC) retrieved full-texts, and two authors (EB and NK) screened the full-texts for inclusion. Any disagreements were resolved by discussion, or reference to a third author (MB, MVD, or CDM). The selection process was recorded in sufficient detail to complete a PRISMA flow diagram (see Figure 1) and a list of excluded (full-text) studies with reasons for exclusions (Supplementary Table S1).

### Data extraction

A data extraction form was used for study characteristics and outcome data, which was piloted on two studies in the review. Two authors extracted the following data from included studies:

- Methods: study authors, location, study design, duration of follow-up
- Participants: *N*, age (mean or median, range or standard deviation), conditions, recent antibiotic use
- Interventions: Type of TH consultation (video, phone, mixed, online), duration, who provided it, training, previous experience
- Primary and secondary outcomes: indication for antibiotics, antibiotic prescribing rate, AE, number of follow-up visits, number of tests requested or samples sent to the laboratory, hospitalisation, antibiotic resistance (if measured in a follow-up visit)

### Assessment of risk of bias

Two authors (MB, and EB or NK) independently assessed the risk of bias for RCTs using the Cochrane Risk of Bias tool^13^ and for observational studies using ROBINS-I.^14^ Owing to the lack of comprehensive manuals, the Newcastle–Ottawa Scale was not used as initially reported in the protocol, which meant that the tool instructions could be interpreted differently by different assessors. All disagreements were resolved by discussion.

### Measurement of effect and data synthesis

Review Manager (version 5.4) was used to calculate the treatment effect. Odds ratios (ORs) were used for dichotomous outcomes reporting the number of patients with an event (for example, antibiotic prescribing). Meta-analyses were only undertaken when meaningful (that is, when ≥2 studies or comparisons reported the same outcome). In anticipation of considerable heterogeneity, a random effects model was used.

In analysis, RCTs were separated from observational studies (for example, cross-sectional studies). Analysis was split by reported conditions (for example, sinusitis, bronchitis). No studies reported the severity of the condition, and so this subgroup analysis was not performed.

Where possible, the individual was used as the unit of analysis. However, data on the number of individuals with primary and secondary outcomes of interest were not available. The information was extracted as it was presented; for example, the number of antibiotic prescriptions for all encounters or visits in each group.

The authors of all included cross-sectional studies were contacted to obtain data of antibiotic prescribing in previous years, to control for any trend of change in antibiotic prescribing. However, the responding authors stated they were unable to provide this for a variety of reasons.

The I^2^ statistic was used to measure heterogeneity. As only one trial was included, a funnel plot was not created.

## Results

### Search results

The searches across three databases yielded 650 records. A backwards (cited) and forwards (citing) citation analysis yielded an additional 433 records. The clinical registry search returned 19 records, and the preprint search via Europe PMC returned an additional 150, resulting in a total of 1067 records to screen after de-duplication. After title and abstract screening, 1011 records were excluded and 56 records were obtained for full-text screening. Thirteen studies were included in the qualitative synthesis and the meta-analysis (Figure 1). See Supplementary Table S1 for a full list of excluded studies with reasons for exclusion.

### Study characteristics

Of the 13 included studies^15–27^ (Supplementary Table S2), all except two were conducted in the US. They comprised 11 cross-sectional studies,^15–17,20–27^ one retrospective before–after study,^19^ and just one RCT.^18^ Nine studies reported antibiotic prescribing for respiratory infections only, two studies provided data for all acute infections (respiratory, urinary, and skin and soft tissue infections), one for both urinary and respiratory infections, and one for urinary infections only. No studies were found that reported on antibiotic prescribing in TH versus F2F consultations for skin and soft tissue infections. The type of TH consultations varied: five studies reported the use of mixed phone and video consultation, four reported phone-only consultations, two reported video consultations, and in two studies the mode was not clearly reported.

### Risk of bias assessment

For the only RCT identified, the Cochrane risk of bias tool was used to assess the risk of bias.^18^ The overall risk of bias was generally unclear. Blinding of the patients and healthcare providers was not possible. Random sequence generation, allocation concealment, blinding of outcome assessment, and the conflict-of-interest statement were all unclear, owing to inadequate reporting in the trial. No evidence was found of incomplete outcome data or selective reporting of outcomes. The study funding was reported.

The ROBINS-I tool found that the remaining 12 studies^15–17,19–27^ were mostly of moderate or serious risk of bias (Table 1). Owing to the study designs, most studies were considered at serious risk of confounding, unless the study authors reported an appropriate analysis method used to adjust for important baseline confounding factors such as age, severity of infection, and any reported comorbidities. Most studies had serious bias for the selection of participants, as patients with less severe infections may differentially choose a mode of consultation (TH rather than F2F). No information was available for the reporting of missing data or selection of the reported results (no available protocols). The included studies had moderate or serious risk of bias in classification of interventions and reported deviations from intended interventions. Measurement of outcomes was rated ‘moderate’ for all studies.

**Table 1.** Risk of bias of included observational studies using ROBINS-I

**Reference**	**Bias owing to confounding**	**Bias in selection of participants into the study**	**Bias in** **classification of interventions**	**Bias owing to** **deviations from intended interventions**	**Bias owing to missing data**	**Bias in** **measurement of outcomes**	**Bias in selection of the reported results**	**Overall risk of bias**
Uscher-Pines (US, 2016)^27^	Serious	Moderate	Moderate	Serious	No available information	Moderate	No available information	Moderate
Gordon (US, 2017)^16^	Serious	Moderate	Moderate	Moderate	No available information	Moderate	No available information	Moderate
Shi (US, 2018)^25^	Moderate	Serious	Moderate	Moderate	No available information	Moderate	No available information	Moderate
Davis (US, 2019)^15^	Serious	Moderate	Moderate	Serious	No available information	Moderate	No available information	Serious
Halpren-Ruder (US, 2019)^17^	Serious	Serious	Moderate	Moderate	No available information	Moderate	No available information	Moderate
Ray (US, 2019)^24^	Moderate	Serious	Moderate	Moderate	No available information	Moderate	No available information	Moderate
Miller (US, 2020)^19^	Serious	Moderate	Moderate	Serious	No available information	Moderate	No available information	Moderate
Murray (US, 2020)^20^	Serious	Serious	Moderate	Serious	No available information	Moderate	No available information	Serious
Penza (US, 2020 A)^22^	Serious	Serious	Serious	Serious	No available information	Moderate	No available information	Serious
Penza (US, 2020 B)^23^	Serious	Serious	Serious	Serious	No available information	Moderate	No available information	Serious
Stenehjem (US, 2020)^26^	Serious	Serious	Moderate	Serious	Moderate	Moderate	No available information	Moderate
Norden (US, 2020)^21^	Serious	Serious	Serious	Serious	No available information	Moderate	No available information	Serious

### Primary outcome: antibiotic prescribing

#### RCTs (*n* = 1)

**Figure 2.**
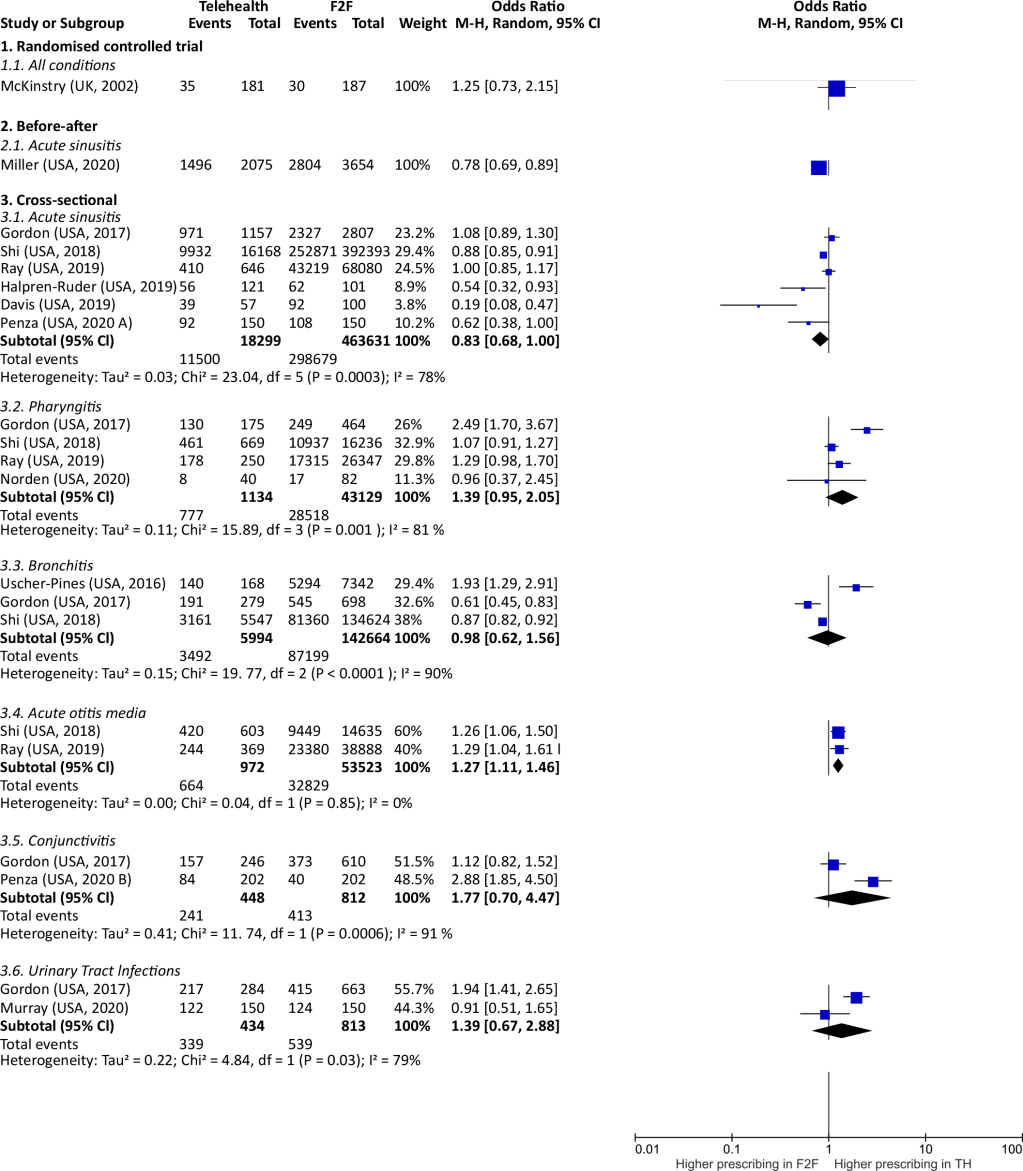
Antibiotic prescribing in synchronous TH compared with F2F consultations

Only one small trial investigated the difference in antibiotic prescribing between patients requesting same-day appointments managed by F2F consultation (*n* = 187) compared with telephone consultation (*n* = 180).^18^ There was more, but not significant, antibiotic prescribing in the TH group compared with F2F consultations (OR = 1.25, 95% confidence intervals [CI] = 0.73 to 2.15) (Figure 2).

#### Before–after study (*n* = 1)

One study examined antibiotic prescribing patterns after the transition to TH visits due to the COVID-19 pandemic, and compared it with the previous F2F visits for acute rhinosinusitis.^19^ There was significantly less antibiotic prescribing in TH consultations (OR = 0.78, 95% CI = 0.69 to 0.89) (Figure 2).

#### Cross-sectional studies (*n* = 11)

Comparison of TH consultations with F2F in cross-sectional studies was subgrouped into the type of reported condition, to reduce confounding of type of consultation by condition (Figure 2).

#### Acute sinusitis (*n* = 6)

There was higher, but not significant, antibiotic prescribing in the F2F group (OR = 0.83, 95% CI = 0.68 to 1.00). Heterogeneity was high (78%).

#### Pharyngitis (*n* = 4)

There was higher, but not significant, antibiotic prescribing in the TH group (OR = 1.4, 95% CI = 0.95 to 2.1). Heterogeneity was high (81%).

#### Bronchitis (*n* = 3)

There was no significant difference in antibiotic prescribing for patients with bronchitis (OR = 0.98, 95% CI = 0.6 to 1.6). Heterogeneity was high (90%).

#### Acute otitis media (*n* = 2)

There was significantly more antibiotic prescribing for patients with acute otitis media in TH consultations (OR = 1.3, 95% CI = 1.11 to 1.46), with no heterogeneity.

#### Conjunctivitis (*n* = 2)

There was higher, but not significant, antibiotic prescribing in the TH group (OR = 1.8, 95% CI = 0.7 to 4.5). Heterogeneity was high (91%).

#### Urinary tract infections (*n* = 2)

There was higher, but not significant, antibiotic prescribing in the TH group (OR = 1.4, 95% CI = 0.7 to 2.9). Heterogeneity was high (79%).

### Secondary outcomes

#### Diagnostic tests performed

Table 2 shows the reported diagnostic tests performed after each type of consultation from six studies. Generally, there are fewer diagnostic tests performed with TH consultations compared with F2F. One study reported the percentage of adults who were diagnosed with pharyngitis and received an appropriate group A Streptococcus (strep) test to confirm the diagnosis.^27^ The F2F group (*n* = 2297, 49.5%) performed better than the TH group (*n* = 4, 3.4%) on appropriate testing for pharyngitis.

**Table 2.** Diagnostic test performed

**Study ID**	**Diagnostic tests requested**	**Condition**	**TH group** ***N* (%**)	**F2F group** ***N* (%**)	**Reported *P* value^a^**
**RCT**					
**McKinstry (UK, 2002**)^18^	Not specified blood test	All conditions	8 (4%)	10 (5%)	Not reported
	Not specified urine test		6 (3%)	8 (4%)	
	X-ray		1 (0.6%)	5 (3%)	
**Cross-sectional studies**					
**Gordon (US, 2017)^b^** ^16^	Not specified lab tests	UTI	85 (20.6%)	1095 (88.4%)	**<** 0.001*
		Pharyngitis	45 (15.8%)	627 (73.5%)	**<** 0.001*
		Sinusitis	185 (11%)	1302 (25.7%)	**<** 0.001*
		Bronchitis	40 (10.1%)	308 (25.8%)	**<** 0.001*
	Not specified Imaging	Cough	18 (11.4)	111 (23.5)	0.001*
		Bronchitis	34 (8.6%)	212 (17.8%)	**<** 0.001*
		UTI	34 (8.2%)	227 (18.3%)	**<** 0.001*
		URI	69 (8.1%)	236 (9.3%)	0.31
		Sinusitis	90 (5.3%)	497 (9.8%)	**<** 0.001*
**Murray (US, 2020)^c^** ^20^	Urinalysis/dip stick	UTI	8 (5%)	140 (93%)	**<** 0.0001*
	Urine culture		11 (7%)	31 (21%)	**<** 0.001*
**Norden (US, 2020)^d^** ^21^	Not specified lab tests	Pharyngitis	0.125	0.207	0.55
		URI excluding pharyngitis	0.023	0.129	0.096
		Otitis media	0.250	0.107	0.60
**Ray (US, 2019**)^24^	Strep test	Streptococcal Pharyngitis	7 (1%)	10 878 (67%)	Not reported
**Shi (US, 2018**)^25^	Strep test	Streptococcal Pharyngitis	9 (4%)	17 818 (68%)	Not reported

*Bold and asterisked *P* values indicate statistical significance.

a χ^2^ test.

b Tests were conducted within 21 days of index visit for all conditions.

c Tests were conducted at initial encounter.

d Average numbers of labs ordered.

URI upper respiratory tract infections UTI urinary tract infections TH telehealth F2F face-to-face

#### Follow-up visits

Seven studies provided results of follow-up visits (See Table 3). In general, patients who were initially evaluated through phone contact were more likely to receive follow up. The studies show different follow-up time points.

**Table 3.** Follow-up characteristics by initial encounter type

**Study ID**	**Follow-up visits within**	**Condition**	**TH group**	**F2F**
**Number of follow-up visits**	**%**	**Number of follow-up visits**	**%**
Ray (US, 2019)^24^	2 days	ARI	226	5	5875	1
	21 days		525	11	45 629	9
Shi (US, 2018)^25^	2 days	ARI	1165	3	4713	0.5
	21 days		3884	10	56 557	6
Gordon (US, 2017)^16^	21 days	All conditions	1302	28	3900	28
Murray (US, 2020)^20^	Same day as initial encounter	UTI	15	10	6	9
	30 days		47	31	39	26
Penza (US, 2020 A)^22^	Same day as initial encounter	Sinusitis	26	49	1	5
	30 days		53	35	21	14
Penza (US, 2020 B)^23^	14 days	Conjunctivitis	92	46	15	7
Norden (US, 2020)^21^	1 day	Pharyngitis	Not reported	40	Not reported	21
		ARI		7		2
		Otitis media		13		7
	3 days	Pharyngitis	Not reported	53	Not reported	28
		ARI		14		9
		Otitis media		13		14

TH telehealth F2F face-to-face ARI acute respiratory infection

#### AE

One study reported no statistically significant difference in the reported AE as evaluated by diagnosis of pyelonephritis within 30-day follow-up duration for patients with urinary tract infections.^20^ The study reported no hospitalisation or sepsis in any patients for either F2F and TH encounters (Supplementary Table S3).

## Discussion

### Summary

This review identified only one RCT that assessed the impact of TH compared with F2F consultations on antibiotic prescribing, which found a non-significant 25% relative increase. Most studies were observational and did not control well for confounding, and therefore were prone to bias. Pooling observational studies did not show a consistent pattern when analysed for specific infections. For instance, antibiotic prescribing for acute sinusitis may be higher in F2F consultation, and for pharyngitis, higher in TH. However, many effect estimates do not reach statistical significance, and the significant heterogeneity suggests methodological issues, rather than clinical differences, within the included studies.

### Comparison with existing literature

The general finding of this study is broadly consistent with the systematic review by Han *et al*,^9^ which concluded there was insufficient evidence that TH consulting has a significant impact on antibiotic prescribing in primary care. In that review, however, the observational studies were pooled and the impact in consultations concerning specific infections was explored. The results show a more diverse picture than can make clinical sense. The two cross-sectional studies that assessed prescribing for acute otitis media^24,25^ both found that antibiotics are more likely to be prescribed in TH consultations. Perhaps the clinician’s inability to examine the ear via remote consultations means that they are more inclined to antibiotic prescribing, especially under parental pressure.^28^

### Strengths and limitations

This review’s main strength is the rigour of its methods and analysis; the extensive search is unlikely to have missed important studies, and the detailed synthesis of the results by study design and by condition has made best use of the available published research. However, there are also several weaknesses. The paucity of studies with adequate control of confounding, the wide heterogeneity (both of clinical conditions and results), and the imprecision of the results means that there is no single reliable message to take away from this research.

### Implications for research

It is important to note there are different modalities of TH (that is, with or without video) which may impact the inclination to prescribe. Also, the link with clinical outcomes and patient satisfaction deserves further exploration.^29^ In situations like the COVID-19 pandemic, synchronous TH consultations have ensured patients’ access to primary care services and changed the landscape of service delivery for good.^30^ Therefore, better understanding of how prescribing adapts to exceptional circumstances is critical for antimicrobial stewardship.

While there is insufficient evidence to draw strong conclusions about the rate of antibiotic prescribing in TH compared with the usual F2F consultations, there are some concerns. The impact appears to vary between conditions, but the findings suggest more conditions saw increases in antibiotic prescribing than reductions. For example, if patients with acute respiratory infections all chose to consult via TH, then the antibiotic prescriptions for TH would be greater than for F2F (and the reverse would be true for those patients who selectively chose F2F consultation). Furthermore, TH may change the diagnostic process because of the limitations on physical examination. Given the impact of any increased antibiotic use on the development of antibiotic resistance,^31^ this clearly suggests more studies need to be undertaken with better design: either randomised trials, or at least controlled before–after studies. To study prescribing change at population level, the ideal study process would be to compare the change in antibiotics when a blend of TH and F2F consultations are introduced with the change when F2F is retained. In a situation where randomisation of practices is not possible, confounding might be adjusted for by using the pre-change level of antibiotic prescribing, and ideally for any trends using a series of time points before the change. If the suggestion is that in some diagnoses more antibiotics are prescribed in F2F consultations, then further research to understand amelioration will become urgent.

## Trial registration number

PROSPERO registration number CRD42021239164
